# Supplementary material for: Modeling assortative mating and genetic similarities between partners, siblings, and in-laws
Source: Nat Commun. 2022 Mar 1;13:1108. doi: 10.1038/s41467-022-28774-y (PMC8888605; doi:10.1038/s41467-022-28774-y)
Supplement: Supplementary file 1 — Supplementary information [file 41467_2022_28774_MOESM1_ESM.pdf]

**SUPPLEMENTARY INFORMATION**

# Modeling assortative mating and genetic similarities between partners, siblings, and in-laws

Fartein Ask Torvik, Espen Moen Eilertsen, Laurie J. Hannigan, Rosa Cheesman, Laurence J. Howe, Per Magnus, Ted Reichborn-Kjennerud, Ole A. Andreassen, Pål Njølstad, Alexandra Havdahl, Eivind Ystrom

Corresponding Author: Fartein Ask Torvik, fartein.ask.torvik@fhi.no

| | |
|---|---|
| **Supplementary Methods 1. MoBa genotyping, imputation, and quality control** | S2 |
| Supplementary Table 1 | S3 |
| **Supplementary Methods 2. The Correlation in Genetic Signal (rGenSi) model: description and simulation** | S4 |
| Supplementary Fig. 1 | S4 |
| Supplementary Fig. 2 | S6 |
| Supplementary Table 2 | S6 |
| Supplementary Fig. 3 | S7 |
| Supplementary Fig. 4 | S8 |
| Supplementary Fig. 5 | S9 |
| Supplementary Table 3 | S9 |
| Supplementary Fig. 6 | S10 |
| Supplementary Table 4 | S11 |
| **Supplementary Note 1. Model fitting of the rGenSi models** | S13 |
| Supplementary Table 5 | S15 |
| Supplementary Table 6 | S16 |
| **Supplementary Note 2. Polygenic scoring method, adjustment for principal components, and sensitivity analyses using different thresholds** | S17 |
| Supplementary Table 7 | S17 |
| Supplementary Table 8 | S18 |
| Supplementary Table 9 | S19 |
| Supplementary Fig. 7 | S20 |
| Supplementary Fig. 8 | S21 |
| **Supplementary References** | S22 |

**Supplementary Methods 1. MoBa genotyping, imputation, and quality control**

The current MoBa genomic dataset comprises imputed genotype data for 98,110 individuals (~32,000 parent-offspring trios), derived from ten batches of participants, who make up four study cohorts. Within each batch, pre-imputation quality control was carried out separately for parent and offspring genetic data. Quality control exclusion criteria for individuals were: genotyping call rate <95%, or autosomal heterozygosity >4 standard deviations from the sample mean. Quality control exclusion criteria for SNPs (single nucleotide polymorphisms) were: ambiguous (A / T and C / G), genotyping call rate <98%, minor allele frequency <1%, or Hardy-Weinberg equilibrium P-value <1 × 10-6. Population stratification was assessed, using the HapMap phase 3 release 3 as a reference, by principal component analysis using EIGENSTRAT version 6.1.4. Visual inspection identified a homogenous population and individuals of non-European ancestries were removed based on principal component analysis of markers overlapping with available HapMap markers. The parent and offspring datasets were then merged into one dataset per genotyping batch, keeping only the SNPs that passed quality control in both datasets. Phasing was conducted using Shapeit2 release 837 and the duoHMM approach was used to account for the pedigree structure. Imputation was conducted using the Haplotype reference consortium (HRC) release 1-1 as the genetic reference panel. The Sanger Imputation Server was used to perform the imputation with the Positional Burrows-Wheeler Transform (PBWT). The phasing and imputation were conducted separately for each genotyping batch. Supplementary Table 1 contains details of the numbers of SNPs and individuals in each batch. More detailed information about the cohorts, quality control and imputation can be found at https://github.com/folkehelseinstituttet/mobagen.

We conducted post-imputation quality control, retaining SNPs meeting the following criteria: INFO≥ 0.8 in all batches, non-duplicated (by position or name), call rate >98%, MAF>1%, HWE p<0.001, not associated with genotyping batch at the genome-wide level, and not causing a mendelian error. We removed individuals with the following criteria: heterozygosity outliers (F-het +/- 0.2), call rate <98%, reported sex mismatching SNP-based sex, duplicates (identified using plink's --genome command as having pihat>=0.98, and distinguished from MZ twins through linkage to unique IDs in the population register, plus age, sex, and kinship information within MoBa), individuals with excessive numbers of close relatives (cryptic relatedness) and mendelian errors. To identify a core sample of individuals with European ancestries, we conducted a principal component analysis using the 1000 Genomes reference; thresholds for exclusion of outliers were based on visual inspection of a plot of principal components 1 and 2. The final numbers of individuals and SNPs passing quality control were 93,582 and 6,797,215, respectively.

**Supplementary Table 1.** MoBa genotyping and imputation information

| Cohort | Genotyping chip | Genotyping center | Cohort design | N SNPs | | | N individuals | | |
|---|---|---|---|---|---|---|---|---|---|
| | | | | **Genotyped** | **Used for imputation** | **Imputed** | **Sent** | **Genotyped** | **Imputed** |
| Harvest | Illumina HumanCoreExome12v1.1 | Genomics core facility, Trondheim, Norway | Trios | 542,585 | | | | 1,692 | |
| | | | | 542,585 | 384,406 | 39,975,206 | 20,668 | 18,972 | 20,231 |
| | Illumina HumanCoreExome24v1.0 | | | 547,644 | 395,736 | 39,963,876 | 12,874 | 12,874 | 12,757 |
| Rotterdam | Illumina Global Screening Array MD v.1.0 | ERASMUS MC, Rotterdam, Netherlands | | 692,367 | 567,903 | 39,791,709 | 27,000 | 17,949 | 17,742 |
| | Illumina Global Screening Array MD v.1.0 | | | 692,338 | 571,759 | 39,787,853 | | 9,041 | 8,959 |
| Norment | Illumina Global Screening Array MD v.01.0 + 50k custom content (OmniExpress overlap) | deCODE Genetics, Rekjavik, Iceland | Trios | 693,143 | 588,807 | 39,770,805 | 9,841 | 9,632 | 9,628 |
| | Illumina HumanOmniExpress-24v1.0 | | Unrelated parents | 708,882 | 665,648 | 39,693,964 | 6,040 | 2,976 | 2,851 |
| | Illumina HumanOmniExpress-24v1.0 | | | 710,146 | 667,978 | 39,691,634 | | 2,983 | 2,983 |
| | Illumina InfiniumOmniExpress-24v1.2 | | Trios | 712,628 | 683,577 | 39,676,035 | 19,611 | 17,730 | 17,730 |
| ADHD | Illumina InfiniumOmniExpress-24v1.2 | | Case trios and singleton controls | 713,599 | 670,860 | 39,688,752 | 5,790 | 5,410 | 5,229 |
| **MoBa Genetics Release 1** | | | | | | | **101,824** | **99,259** | **98,110** |

## Supplementary Methods 2. The Correlation in Genetic Signal (rGenSi) model: description and simulation

*The basic rGenSi model*

The basic version of the correlation in genetic signal model (rGenSi) is shown in Supplementary Fig. 1. The model uses data on phenotypes and polygenic scores (PGS) from four adult individuals: Two siblings and their partners. Hence, it includes siblings, partners, siblings-in-law (in-laws for short), and co-siblings-in-law (co-in-laws for short). The model takes eight variables as input and reduces the 8x8 correlation matrix to four estimated parameters, *s*, *h*, *m*, and $r_s$. It includes latent genetic factors that influence the phenotype and the polygenic score of each individual. It captures the variance common to the phenotype and the polygenic score and thereby represents the genetic signal of interest. The correlation between an individual's phenotype and polygenic score is decomposed into *s*, which is an estimate of the genetic signal in the polygenic score, and *h*, which is the influence of genetic factors on the phenotype. Squared, $s^2$ is the proportion of variance in the polygenic score explained by the latent genetic signal, and $h^2$ is an estimate of the heritability. The correlation between the polygenic score and the phenotype for an individual is $s * h$. Residual variance additionally influences phenotypes and polygenic scores. These are not freely estimated as the variance is fixed to 1 in all variables. With data on individuals, *s* and *h* cannot be freely estimated. This is possible when including data on relatives.

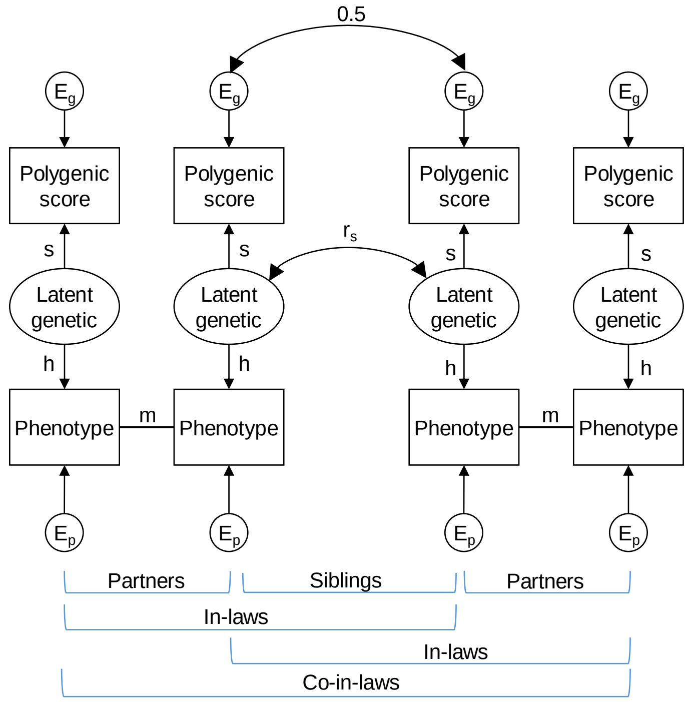


**Supplementary Fig. 1. The basic rGenSi model**

The model assumes that partners assort directly on the phenotype; hence partners are linked by the co-path *m*. All partner similarity goes through this co-path. Co-paths are an extension of conventional path tracing rules introduced by Cloninger [1]. A summary of rules concerning co-paths are found in Balbona et al. [2]. In short, co-paths have no arrows (direction), and connect other valid chains of paths. They contribute to covariance between variables, but not to variance. This is useful when analyzing assortative mating because the assortment of partners does not change their phenotypes but leads to an association. The basic rGenSi model assumes that the phenotypes on which the assortment takes place are perfectly measured and included. It also assumes that there is no shared environmental variance between siblings. Hence, in this model, siblings can only resemble each other for genetic reasons. The correlation in the genetic signal between siblings is estimated in this model

as $r_s$. Under random assortment, the genetic correlation between siblings is expected to be 0.50. However, it can rise above this value due to positive assortment in previous generations. In addition, the polygenic scores can be influenced by factors not related to the phenotype, which are expected to correlate 0.50 between siblings. The correlation between the different individuals' latent genetic factors can be deduced by path tracing rules including co-paths. Hence, the correlation between siblings' genetic signals is $r_s$. The correlation between partners' genetic signal is $h * m * h = mh^2$, the correlation between in-laws' genetic signal is $h * m * h * r_s = r_s mh^2$, and the correlation between co-in-laws' genetic signal is $h * m * h * r_s * h * m * h = r_s m^2 h^4$. The correlation between one's polygenic score and one's own phenotype is $s * h$, whereas the correlation between one's polygenic score and one's partner's phenotype is $s * h * m$. Hence, $m$ can be estimated by dividing the cross-partner phenotype-polygenic correlation ($s * h * m$) with the within individual phenotype-polygenic correlation ($s * h$). (Robinson et al. derived the phenotypic correlation in an equivalently way by regressing a phenotype on the partner's genetic predictor [3].) Other correlations between observed phenotypes or polygenic scores can similarly be deduced with path tracing rules. For instance, the correlation between partners' phenotypes is $m$, whereas the correlation between co-in-laws' polygenic scores is $s * h * m * h * r_s * h * m * h * s = r_s m^2 h^4 s^2$. As we will show later with empirical data, this model fits excellent for height.

*Simulation of the basic rGenSi model*

To test whether this model can accurately estimate $s$, $h$, $m$, and $r_s$, we ran 1,000 simulations with a sample size of 50,000 individuals and 1,000 simulations with a sample size of 1,000 individuals. Each extended family consisted of four individuals, so for the sample size of 1,000 individuals, there were 250 extended families which included 250 complete pairs of siblings, 500 complete pairs of partners, 500 complete pairs of in-laws, and 250 complete pairs of co-in-laws. In each simulation, we drew $s^2$ and $h^2$ from a uniform distribution ranging from 0.20 to 1.00 and $m$ from a uniform distribution ranging from 0.00 to 1.00. We simulated 100 genetic variants related to a phenotype for a founder population of the stated sample size who mated with each other and produced offspring for 10 generations. The polygenic score was a partial measure of the true underlying genetic signal and included a fraction of the causal SNPs equal to $s^2$. For instance, 50 of these 100 genetic variants were included in the polygenic score if $s^2 = 0.50$ and $s = 0.71$. Within each generation, individuals within each sex were ranked according to the sum of their phenotype and a random component representing "assortment noise". The "assortment noise" was sampled from a normal distribution with mean zero and variance $(1 – m) / m$, where $m$ is the correlation among partners. Individuals were then mated according to their rank, i.e., the highest scoring within sex 1 with the highest scoring within sex 2 and so on. The resulting partner correlations were stable over generations. Siblings in the founder generation were the product of random mating and shared on average half of the genetic variants; if estimated in the first generation, $r_s$ would therefore be 0.50, but this rises over generations with assortment. The expected value of $r_s$ can be derived mathematically from $m$ and $h$ and can also be observed directly in the simulated data. In the simulated data, the genetic factor is not a latent variable but an observed variable including all causal genetic variants, also those excluded from the polygenic score. It is not included in the model but allows one to check if the model correctly estimates the genetic correlations using the imperfect polygenic scores.

In the 10$^{th}$ generation, we estimated the rGenSi model based on the four simulated phenotypes and the four simulated polygenic scores. This process was repeated 1,000 times for each sample size with random values of $s$, $h$, and $m$, as described above. Supplementary Fig. 2 shows the correspondence between simulated true values and the corresponding values estimated in the rGenSi model. Supplementary Table 2 shows the means, SDs, and medians of the true and the estimated values. The match between true and estimated values was excellent with 50,000 simulated individuals. With 1,000 simulated individuals, the match was good in a majority of datasets. In conclusion, the model can reconstruct the relevant parameters in realistic scenarios.

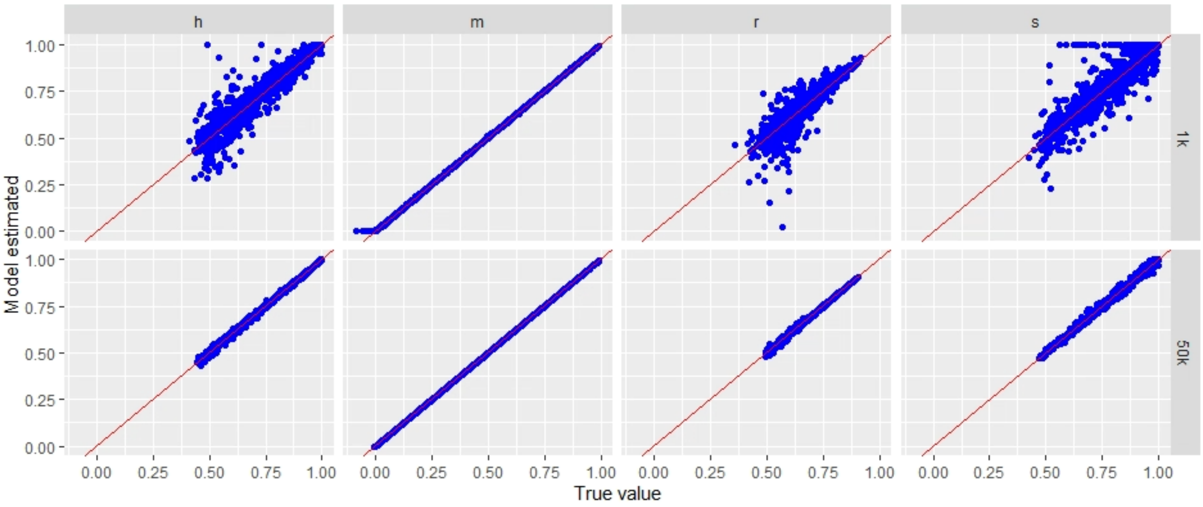


**Supplementary Fig. 2. Simulation of the basic rGenSi model.** Comparison of true and observed values for *h*, *s*, *m*, and $r_s$. 1,000 repetitions with a sample size of 50,000 and 1,000 repetitions with a sample size of 1,000.

**Supplementary Table 2**. Means, standard deviations (SD), and medians of true and observed values (estimated with the rGenSi model) with a sample size of 1,000 and 50,000, excluding *a* and *c*.

| **N=1,000** | | | | | | |
|---|---|---|---|---|---|---|
| Parameter | Mean of true values | Mean of observed values | SD of true values | SD of observed values | Median of true values | Median of observed values |
| h | 0.759 | 0.761 | 0.151 | 0.160 | 0.775 | 0.782 |
| m | 0.460 | 0.462 | 0.282 | 0.281 | 0.442 | 0.444 |
| r | 0.630 | 0.630 | 0.101 | 0.116 | 0.618 | 0.622 |
| s | 0.790 | 0.794 | 0.146 | 0.158 | 0.815 | 0.814 |
| **N=50,000** | | | | | | |
| Parameter | Mean of true values | Mean of observed values | SD of true values | SD of observed values | Median of true values | Median of observed values |
| h | 0.762 | 0.762 | 0.158 | 0.158 | 0.776 | 0.776 |
| m | 0.486 | 0.486 | 0.280 | 0.280 | 0.480 | 0.480 |
| r | 0.642 | 0.642 | 0.100 | 0.100 | 0.618 | 0.618 |
| s | 0.806 | 0.805 | 0.140 | 0.140 | 0.825 | 0.828 |

*The rGenSi model with mating on a latent phenotype and influences of shared environment*

Two additional parameters may be added to the rGenSi model. This version of the model is shown in Supplementary Fig. 3. The first parameter is *a* (alpha), or the correlation between the measured phenotype and the one on which mating takes place. The assortment between partners may not take place on the measured variable. Like the polygenic scores, the phenotypic variable could be imperfectly measured, or it could be that the assortment takes place on another variable that correlates with the one we have observed (indirect or secondary assortment). Mating on a latent phenotype related to the observed phenotype resembles the *Cascade model* [4]. For instance, current depression may not be the basis of assortment; rather, individuals may assort based on their tendency to be depressed or how depressed they were when they met. Likewise, educational attainment may not per se be the factor underlying assortment; related variables, such as personality and cognitive abilities, could be relevant. By adding one parameter to the rGenSi model, this can be estimated. This parameter is the association between the observed phenotype and the unobserved

and potentially composite phenotype on which mating occurs. The model is shown in Supplementary Fig. 3. The *a* parameter can be identified because it can reasonably be assumed to be equal in all relatives. Path tracing shows that adding this parameter reduces correlations between all relationship types to the same degree (all correlations between phenotypes are in the basic rGenSi model, but multiplied by $a^2$; the formulas for correlations between polygenic scores are not changed). As we will later show with empirical data, this model fits well for depression, whereas a freely estimated alpha is unnecessary for height (a=1 for height). In this model, *m* is not simply the correlation between two partners' observed phenotypes but derived by fitting the 8x8 correlation structure to this model with 5 freely estimated parameters. In this version of the model, where *a* is freely estimated, the correlation between one's polygenic score and one's own phenotype is $a * s * h$, whereas the correlation between one's polygenic score and one's partner's phenotype is $a * s * h * m$. It is still possible to estimate *m* by dividing the cross-partner phenotype-polygenic correlation with the within individual phenotype-polygenic correlation, as $(a * s * h * m) / (a * s * h) = m$. However, *m* now describes the similarity in the latent phenotype underlying assortment rather than the observed phenotypic similarity. As we will show with empirical data, a freely estimated *a* leads to improved fit for models of depression and educational attainment.

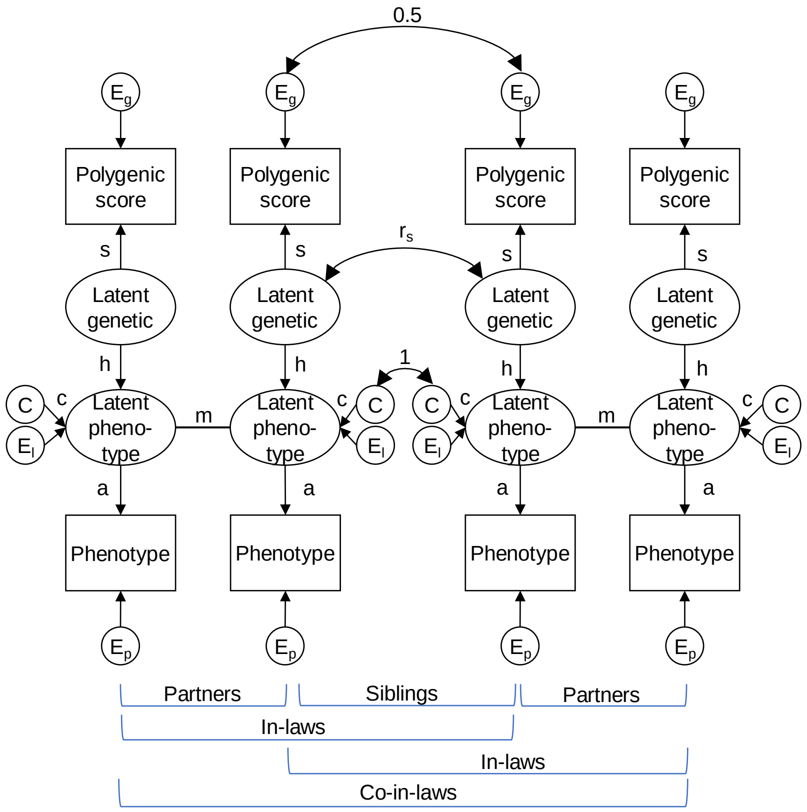


**Supplementary Fig. 3. The full rGenSi model.** The rGenSi model with freely estimated alpha (*a*) and shared environment (*c*).

The second parameter that can be added to the model is effects of shared environment, defined as factors that make siblings similar to each other. Shared environment is added to the model as a factor perfectly shared between siblings that influences both siblings' phenotypes and is unrelated to the genetic factors. Hence, if the siblings' phenotypes correlate higher than expected from the correlation between their polygenic scores and between their polygenic scores and phenotypes, we can expect a positive value of *c*, interpreted as environmental factors that make siblings similar to each other. Note that our estimates of *h* and *c* do not rely on the equal environments assumption. These two parameters, *a* and *c,* may be added independently of each other. The model described in the previous section corresponds to the extended model where *c* has been set equal to 0, and *a* has

been set to 1. As we will show with empirical data, the inclusion of shared environment leads to the best fit for educational attainment.

*Simulation of the full rGenSi model*

To test whether the rGenSi model can estimate *c* and *a* simultaneously as it estimates *s*, *h*, *m*, and $r_s$, we simulated 1,000 new datasets with 50,000 individuals and 1,000 new datasets with 1,000 individuals using process described above. Alpha (*a*) was drawn randomly from a uniform distribution with values from 0.20 to 1.00, whereas $c^2$ was drawn randomly from a uniform distribution ranging from 0.00 to 1.00. If the sum of $c^2$ and $h^2$ exceeded 1.00, both values were redrawn. Otherwise, this simulation followed the same rules as the simulation of the basic rGenSi model, described on page 4 of this supplement. (We drew $s^2$ and $h^2$ from uniform distributions ranging from 0.20 to 1.00 and *m* from a uniform distribution ranging from 0.00 to 1.00. In the first generation $r_s$ equals 0.50, but it can rise over generations with assortment, depending on the other parameters. We simulated 100 genetic variants related to a phenotype and assortment for 10 generations before we estimated the rGenSi model.) The correspondence between the true and estimated values is shown in Supplementary Fig. 4.

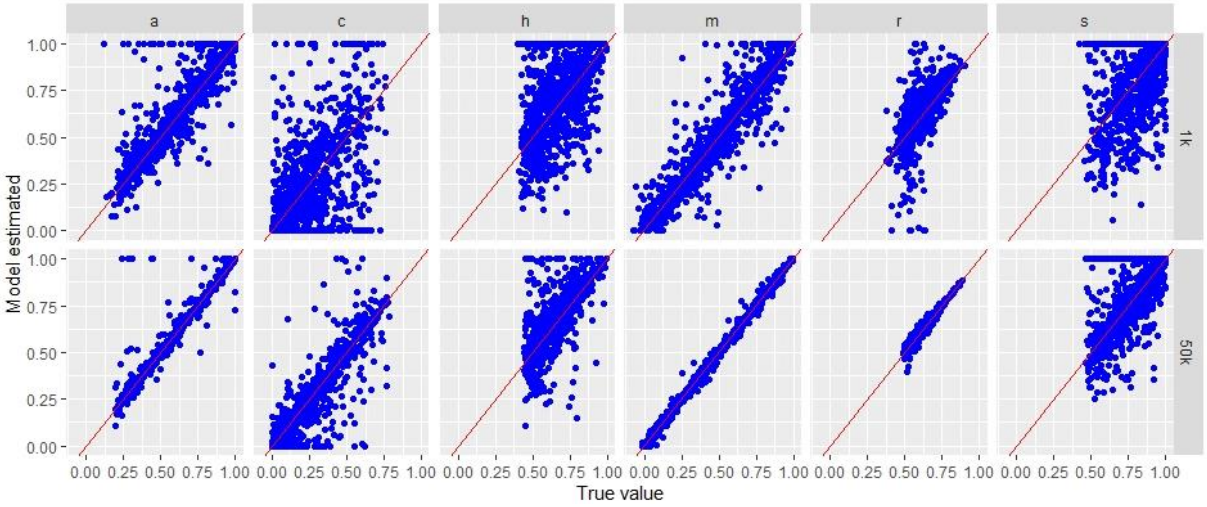


**Supplementary Fig. 4. Simulation of the full rGenSi model.** Comparison of true and observed values for *h*, *s*, *m*, $r_s$, *a*, and *c*. 1,000 repetitions with a sample size of 50,000 and 1,000 repetitions with a sample size of 1,000.

A low *a* value reduces the associations between the observed phenotypes – in the extreme scenario that *a* is close to 0, these would be close to uncorrelated, and it would be near impossible to reconstruct the relations between the mated phenotypes. Therefore, we filtered out the simulations where *a* was > 0.60 and present these in Supplementary Fig. 5. Supplementary Table 3 shows the means, SDs, and medians of the true expected values and estimated values for the corresponding data. Whereas the model with *a* and effects of shared environment is less precise than the more restricted model, it provides reasonable estimates of the six parameters in most cases when the sample size is large. It is noteworthy that it can estimate the partner association in the mated phenotypes (*m*) with high accuracy even when this phenotype is not directly measured. Nevertheless, we recommend using the more restricted model when it has a good fit.

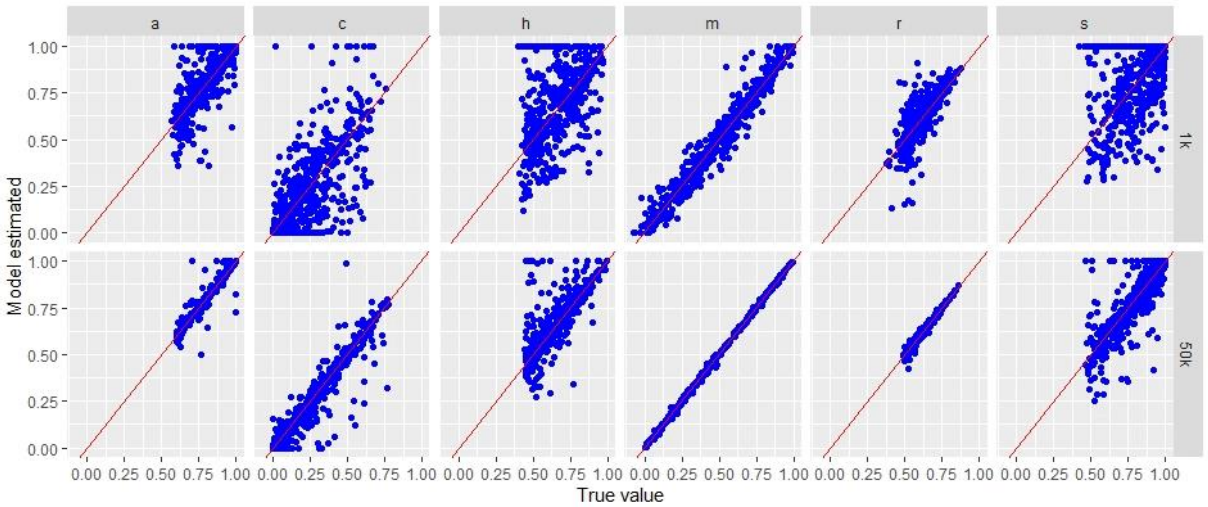


**Supplementary Fig. 5. Simulation of the full rGenSi model filtered on *a* > 0.60.** Comparison of true and observed values for *h*, *s*, *m*, $r_s$, *a*, and *c*. The simulations are the same as in Supplementary Fig. 4 but filtered on *a* > 0.60.

**Supplementary Table 3**. Means, standard deviations (SD), and medians of true and observed values (estimated with the rGenSi model) with a sample size of 1,000 and 50,000, including free estimation of *a* and *c*; excluding *a* < 0.60.

| **N=1,000** | | | | | | |
|---|---|---|---|---|---|---|
| Parameter | Mean of true values | Mean of observed values | SD of true values | SD of observed values | Median of true values | Median of observed values |
| a | 0.798 | 0.803 | 0.115 | 0.142 | 0.800 | 0.817 |
| c | 0.265 | 0.253 | 0.178 | 0.233 | 0.236 | 0.195 |
| h | 0.667 | 0.687 | 0.142 | 0.202 | 0.665 | 0.705 |
| m | 0.480 | 0.485 | 0.270 | 0.275 | 0.486 | 0.468 |
| r | 0.609 | 0.614 | 0.100 | 0.118 | 0.603 | 0.616 |
| s | 0.799 | 0.810 | 0.146 | 0.198 | 0.824 | 0.867 |
| **N=50,000** | | | | | | |
| Parameter | Mean of true values | Mean of observed values | SD of true values | SD of observed values | Median of true values | Median of observed values |
| a | 0.794 | 0.794 | 0.116 | 0.119 | 0.802 | 0.799 |
| c | 0.281 | 0.269 | 0.197 | 0.203 | 0.248 | 0.223 |
| h | 0.660 | 0.677 | 0.138 | 0.156 | 0.654 | 0.674 |
| m | 0.509 | 0.510 | 0.278 | 0.280 | 0.485 | 0.492 |
| r | 0.614 | 0.615 | 0.083 | 0.085 | 0.592 | 0.597 |
| s | 0.794 | 0.786 | 0.147 | 0.168 | 0.833 | 0.822 |

**Testing the assumption that the residual genetic correlation between siblings is 0.50**

The residual component of the polygenic score is defined as not being associated with the phenotype. Therefore, we assumed that it could not be a basis for partner selection and that is should not correlate between partners. Similarly, we assume that this genetic residual was not associated with partner selection in previous generations and thereby assume that the residual genetic correlation between siblings is 0.50. However, this is not statistically necessary to make this assumption. It is also possible to test. If effects of shared environment (*c*) are fixed to a specific value (usually zero), it is possible to freely estimate the correlation between siblings' genetic residuals. In the rGenSi model, it is unfortunately not possible to simultaneously estimate both effects of shared

environment and the correlation between siblings' genetic residuals. The genetic correlation between siblings' residuals was estimated very close to 0.50 in all tested simulations when we fixed *c=0*. (In simulations, we also generated half-siblings sharing 0.25 of genetic factors and a fictitious "worker bee" sibling type sharing 0.75 of genetic factors. In these cases, the correlation between genetic residuals matched the expected 0.25 and 0.75, respectively.) Hence, we conclude that the residual correlation between siblings' polygenic scores can be freely estimated if effects of shared environment (*c*) are not included in the model but that it is 0.50 in realistic scenarios.

*Increase in genetic variance and sibling correlations over generations*
Supplementary Fig. 6 shows how the genetic variance (g_var) and the genetic correlations between siblings increase after 0 to 15 generations of assortative mating when the assortment (phenotypic correlations between partners) is 0.40, and the initial heritability is 0.60. These numbers are roughly similar to those for educational attainment. As can be seen, values close to equilibrium are reached after relatively few generations. For instance, the curves are close to equilibrium after 5 generations, and no increase is seen after 10 generations.

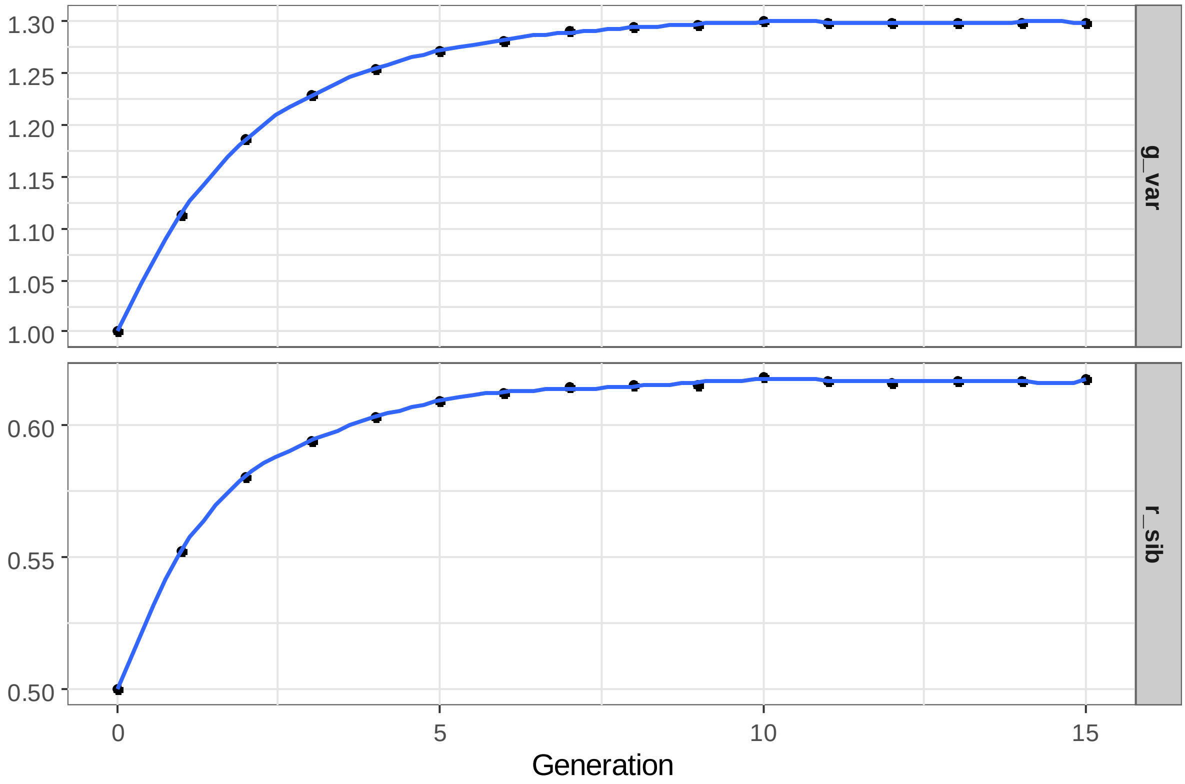


**Supplementary Fig. 6. Genetic variance and covariance over generations.** Genetic variance and genetic sibling correlations after 0 to 15 generations of assortative mating on an initially 60% heritable phenotype with stable assortment of 0.40.

*Testing intergenerational equilibrium with imperfect indicators of genotype and phenotype*
We test whether the genetic variance is in intergenerational equilibrium by comparing genetic correlations between siblings and partners. In intergenerational equilibrium, these can be predicted from one another, as the expected $\widehat{r_{g,sibling}} = \frac{1}{2}(1 + r_{g,partner})$ and $\widehat{r_{g,partner}} = 2r_{g,sibling} - 1$. For instance, if the genetic correlation between partners is 0.20, the genetic correlation between siblings is 0.50+0.20/2=0.60 in equilibrium. The observed and predicted sibling correlations can be compared with the following formula:

$$U = \frac{(r_{g,sibling} - 0.50)}{(\widehat{r_{g,sibling}} - 0.50)}$$

In the first generation, before assortment started, the observed siblings correlation is 0.50; hence U=0.00. In intergenerational equilibrium, the observed and expected correlations should match, and U would have the expected value of 1.00.

The rGenSi model allows for both phenotypes and polygenic scores to be imperfect indicators of the phenomena causing partner similarity. When $a$ is less than 1, the phenotype is an imperfect indicator of the phenotype that is the basis for selection, whereas when $s$ is less than 1, the polygenic score is an imperfect indicator of the relevant genetic factors. When polygenic scores do not include all relevant causal genetic variants ($s$<1), both partner and sibling correlations can be underestimated towards the expected values under no assortment (0.00 for partners and 0.50 for siblings). The genetic correlation between siblings is directly estimated in the model as $r_{g,sibling} = r_s$ and the genetic correlation between partners is derived by path tracing as $r_{g,partner} = hmh$. Hence, $r_{g,sibling}$ and $r_{g,partner}$ are independent of both $s$ and $a$. The genetic correlations between the latent genetic factors should thus not be expected to be affected by the imperfection of measurement in phenotypes or in polygenic scores, and genetic sibling and partner correlations can be used to test intergenerational equilibrium even when $s$<1 and $a$<1. The relative deviation from equilibrium, indicated by U, should be consistent across varying proportions of genetic variance covered by the observed polygenic scores. Thus, one can test intergenerational equilibrium even if the genetic variables are partial, for instance, by using polygenic scores directly. Likewise, the partner and sibling correlations can be used to test whether the genetic variance is in intergenerational equilibrium both under direct and under indirect assortment.

To ensure that this reasoning is correct, we ran simulations similar to those described above. We used a sample size of 50,000. Initial simulations indicated that U was always (close to) 1.00 after 10 generations, even when $a$ and $s$ were <1. This indicates equilibrium. To make the simulations more interesting, each simulation was run for between 1 and 6 generations (drawn from a uniform distribution), so the genetic variance is plausibly not in equilibrium. We set each of $s^2$ and $a$ to 0.90 and to 0.30, giving four combinations of $s^2$ and $a$ for each data set. In each dataset, we estimated U four times. We did this in 100 datasets. The results with the four different combination of values for $a$ and $s$ values were similar, as indicated by Supplementary Table 4. The results were robust to low values of $a$, that is, to indirect selection. A low $s^2$ led to reduced accuracy in the estimations, in particular in combination with a low $a$, but the results were not biased in any particular direction.

**Supplementary Table 4**. Estimating deviations from intergenerational equilibrium with imperfectly measured genetic and phenotypic variables.

| | $s^2$=0.9, a=0.9 | $s^2$=0.3, a=0.9 | $s^2$=0.9, a=0.3 | $s^2$=0.3, a=0.3 |
|---|---|---|---|---|
| Mean U | 0.695 | 0.701 | 0.697 | 0.716 |
| Median U | 0.715 | 0.714 | 0.714 | 0.719 |
| SD U | 0.191 | 0.196 | 0.192 | 0.243 |
| Median absolute difference from $s^2$=0.9, a=0.9 | 0.000 | 0.033 | 0.008 | 0.037 |
| Correlation with $s^2$=0.9, a=0.9 | 1 | 0.930 | 0.993 | 0.749 |
| Correlation with $s^2$=0.3, a=0.9 | 0.930 | 1 | 0.922 | 0.790 |
| Correlation with $s^2$=0.9, a=0.3 | 0.993 | 0.922 | 1 | 0.740 |
| Correlation with $s^2$=0.3, a=0.3 | 0.749 | 0.790 | 0.740 | 1 |

*Partialness versus noise in polygenic scores*

An imperfect correlation between the polygenic score and the genetic signal can be due to partialness of the polygenic score (it does not cover all relevant genetic variants) and due to inclusion of irrelevant genetic factors (noise). These two mechanisms both reduce the correlation between the

latent genetic variable and the polygenic score, estimated as *s*. Additional simulations indicated that *s* is correctly estimated regardless of which these two mechanisms make the polygenic scores imperfect. We have not found a way to distinguish between these in the model while maintaining the other parameters, but as s appears to be correctly estimated, the difference between these two mechanisms is irrelevant to the current research question.

*Scripts*

Scripts for the rGenSi model and the simulations are provided in Supplementary Software 1 and at https://osf.io/v9ybu/.

**Supplementary Note 1. Model fitting of the rGenSi models**

Supplementary Table 5 shows the model fit indices for the rGenSi models for the three phenotypes educational attainment, height, and depression. First, the full rGenSi model (with freely estimated *a* and *c*) is compared to a saturated model to test how well it fits the data. Then, more restricted versions of the rGenSi model are compared to this full model to see if these simpler models can adequately account for the observed correlational structure.

For educational attainment, the AIC value improved (was lower) in the rGenSi model, compared to the saturated model, indicating a better balance between parsimony and complexity. However, restricting *c* to 0, *a* to 1, or both, increased the AIC values, indicating a worse fit. Models with *a* set to 1 had a dramatically worse fit, indicating that the observed educational attainment is not identical to the (composite) phenotype that is the basis of partner selection and for the observed partner resemblance in educational attainment. For height, the model with no effects of shared environment and *a* equal to 1 had the best fit. This indicates that measured height is a basis of partner selection. For depression, the best fitting model had no shared environment but *a* below 1. This indicates that depression assessed at the time of the survey was not alone the basis of partner selection, but rather some highly correlated variable; this could, for example, be risk for depression or maybe manifest depression at the time the partnership was initiated.

In the full model, the genetic partner correlation was 0.37, predicting a genetic sibling correlation of 0.37/2+0.50 = 0.69, which compared well to the estimated sibling correlation of 0.68. Hence, in the full model, we found no deviations from intergenerational equilibrium in the genetic factors for educational attainment (p=0.987). The results would have been different had we based this comparison on the more restricted model. In the most restricted model (with *a* = 1 and *c* = 0), the genetic partner correlation was 0.21, predicting a genetic sibling correlation of 0.21/2+0.50 = 0.61, considerably lower than the observed 0.70 (p-value for deviation from equilibrium = 0.047). Hence, had we not used latent phenotypes, we could have concluded that the genetic variance in educational attainment was not in intergenerational equilibrium, but these models had a worse fit.

The model of educational attainment with no effects of shared environment (model 3) was close in fit to the one that included effects of shared environment (model 2). Three indices indicate that model 2 is the correct one. First, educational attainment is known from previous studies to be influenced by shared environment. Second, model 6, with shared environment and equilibrium, exhibits the overall best fit. Third, model 7 (no shared environment and in equilibrium) has a poor fit and does not correspond with the results showing that the crude polygenic scores are in intergenerational equilibrium (presented next). Therefore, the models with shared environment (model 2 and model 7) fit best not only in terms of statistical fit, but also with previous research and parallel analytic approaches.

Intergenerational equilibrium can also be tested by adding constraints to the estimations of correlations between the polygenic scores. Constraining the correlations between polygenic scores in siblings and partners to intergenerational equilibrium did not indicate deviations for equilibrium for educational attainment (Δ-2LL = 0.55, Δdf=1, p=0.457), height (Δ-2LL = 0.01, Δdf=1, p=0.935), or depression (Δ-2LL = 2.78, Δdf=1, p=0.095).

Supplementary Table 6 shows the model parameters for the four different versions of the rGenSi model and the resulting estimates of genetic correlations between relatives. The most restricted model had the best fit for height and was relatively similar to the best fitting model for depression, with partner correlation and heritability being a bit higher in the model with mating on latent variables. However, for educational attainment, the model that freely estimated *a* had some noticeable differences from the most restricted model. First, in the restricted model, *m* is equal to

the observed correlation between partners, whereas in the model where *a* is estimated freely, *m* is the partner correlation in a latent variable on which partners assort phenotypically. Hence, estimating *m* based on the observed or latent phenotypes leads to different estimates of partner correlations and thus also of genetic partner resemblance. The more restricted models had narrower confidence intervals than the more open models, indicating that the more restricted models should be preferred when they fit the data well.

**Supplementary Table 5**. Model fit statistics for saturated models and rGenSi models with and without freely estimated alpha (a) and shared family environment (c).

| # | Model | ep | minus2LL | df | AIC | Comp-arison | Δ-2LL | Δdf | p |
|---|---|---|---|---|---|---|---|---|---|
| | **Educational attainment** | | | | | | | | |
| 1 | Saturated model | 36 | 323369.72 | 117649 | 88071.72 | - | | | |
| 2 | rGenSi, free a, free c* | 6 | 323415.37 | 117679 | 88057.37 | 1 | 45.65 | 30 | 0.034 |
| 3 | rGenSi, free a, c=0 | 5 | 323417.46 | 117680 | 88057.46 | 2 | 2.10 | 1 | 0.148 |
| 4 | rGenSi, a=1, free c | 5 | 323857.09 | 117680 | 88497.09 | 2 | 441.72 | 1 | 0.000 |
| 5 | rGenSi, a=1, c=0 | 4 | 323859.10 | 117681 | 88497.10 | 2 | 443.73 | 2 | 0.000 |
| 6 | free a, free c, equilibrium** | 6 | 323415.37 | 117680 | 88055.37 | 2 | 0.00 | 1 | 0.987 |
| 7 | free a, c=0, equilibrium | 5 | 323422.89 | 117681 | 88060.89 | 3 | 5.43 | 1 | 0.020 |
| 8 | a=1, free c, equilibrium | 5 | 323863.10 | 117681 | 88501.10 | 4 | 6.02 | 1 | 0.014 |
| 9 | a=1, c=0, equilibrium | 4 | 323863.04 | 117682 | 88499.04 | 5 | 3.94 | 1 | 0.047 |
| | **Height** | | | | | | | | |
| # | Model | ep | minus2LL | df | AIC | Comp-arison | Δ-2LL | Δdf | p |
| 1 | Saturated model | 36 | 273104.29 | 101552 | 70000.29 | - | | | |
| 2 | rGenSi, free a, free c | 6 | 273129.02 | 101582 | 69965.02 | 1 | 24.73 | 30 | 0.738 |
| 3 | rGenSi, free a, c=0 | 5 | 273129.41 | 101583 | 69963.41 | 2 | 0.39 | 1 | 0.533 |
| 4 | rGenSi, a=1, free c | 5 | 273130.31 | 101583 | 69964.31 | 2 | 1.29 | 1 | 0.257 |
| 5 | rGenSi, a=1, c=0* | 4 | 273130.71 | 101584 | 69962.71 | 2 | 1.68 | 2 | 0.431 |
| 6 | free a, free c, equilibrium | 6 | 273129.02 | 101583 | 69963.02 | 2 | 0.00 | 1 | 0.967 |
| 7 | free a, c=0, equilibrium | 5 | 273130.18 | 101584 | 69962.18 | 3 | 0.77 | 1 | 0.380 |
| 8 | a=1, free c, equilibrium | 5 | 273130.33 | 101584 | 69962.33 | 4 | 0.02 | 1 | 0.896 |
| 9 | a=1, c=0, equilibrium** | 4 | 273131.10 | 101585 | 69961.10 | 5 | 0.40 | 1 | 0.528 |
| | **Depression** | | | | | | | | |
| # | Model | ep | minus2LL | df | AIC | Comp-arison | Δ-2LL | Δdf | P |
| 1 | Saturated model | 36 | 323472.47 | 114704 | 94064.47 | - | | | |
| 2 | rGenSi, free a, free c | 6 | 323488.72 | 114734 | 94020.72 | 1 | 16.25 | 30 | 0.981 |
| 3 | rGenSi, free a, c=0* | 5 | 323489.62 | 114735 | 94019.62 | 2 | 0.90 | 1 | 0.342 |
| 4 | rGenSi, a=1, free c | 5 | 323495.50 | 114735 | 94025.50 | 2 | 6.79 | 1 | 0.009 |
| 5 | rGenSi, a=1, c=0 | 4 | 323496.27 | 114736 | 94024.27 | 2 | 7.55 | 2 | 0.023 |
| 6 | free a, free c, equilibrium | 6 | 323491.48 | 114735 | 94021.48 | 2 | 2.76 | 1 | 0.096 |
| 7 | free a, c=0, equilibrium** | 5 | 323491.51 | 114736 | 94019.51 | 3 | 1.89 | 1 | 0.170 |
| 8 | a=1, free c, equilibrium | 5 | 323499.17 | 114736 | 94027.17 | 4 | 3.69 | 1 | 0.056 |
| 9 | a=1, c=0, equilibrium | 4 | 323499.20 | 114737 | 94025.20 | 5 | 2.93 | 1 | 0.087 |

Notes: * best fitting model before adding equilibrium constraint. ** previously best fitting model with equilibrium constraint added.The difference in -2 times log likelihood (Δ-2LL) is asymptotically $\chi^2$-square distributed (with Δdf degrees of freedom), which allows testing for significant differences in $\chi^2$ for nested submodels. If the difference in $\chi^2$ is non-significant, the simpler, more restricted model is preferred. In addition, models with low Akaike Information Criterion are preferred.

**Supplementary Table 6.** Parameters estimated in rGenSi models and genetic correlations between relatives implied from these models.

**Educational attainment**

| Parameter | Full (best) | | | Free a,c=0 | | | a=1, free c | | | a=1, c=0 | | |
|---|---|---|---|---|---|---|---|---|---|---|---|---|
| | est | cilo | cihi | est | cilo | cihi | est | cilo | cihi | est | cilo | cihi |
| s | 0.50 | 0.35 | 0.65 | 0.39 | 0.36 | 0.41 | 0.50 | 0.35 | 0.66 | 0.39 | 0.36 | 0.41 |
| h | 0.73 | 0.52 | 0.95 | 0.96 | 0.90 | 1.01 | 0.56 | 0.39 | 0.73 | 0.73 | 0.68 | 0.78 |
| m | 0.68 | 0.65 | 0.70 | 0.68 | 0.65 | 0.70 | 0.40 | 0.39 | 0.41 | 0.40 | 0.39 | 0.41 |
| r | 0.68 | 0.61 | 0.75 | 0.69 | 0.62 | 0.77 | 0.68 | 0.61 | 0.76 | 0.70 | 0.62 | 0.77 |
| a | 0.77 | 0.75 | 0.78 | 0.77 | 0.75 | 0.78 | | | | | | |
| c | 0.27 | 0.03 | 0.50 | | | | 0.15 | 0.01 | 0.30 | | | |
| Rgpartner | 0.37 | 0.21 | 0.67 | 0.62 | 0.54 | 0.71 | 0.13 | 0.07 | 0.23 | 0.21 | 0.19 | 0.24 |
| Rgsibling | 0.68 | 0.61 | 0.75 | 0.69 | 0.62 | 0.77 | 0.68 | 0.61 | 0.76 | 0.70 | 0.62 | 0.77 |
| Rginlaw | 0.25 | 0.14 | 0.46 | 0.43 | 0.39 | 0.47 | 0.09 | 0.05 | 0.16 | 0.15 | 0.13 | 0.16 |
| Rgcoinlaw | 0.09 | 0.03 | 0.31 | 0.27 | 0.22 | 0.33 | 0.01 | 0.00 | 0.04 | 0.03 | 0.03 | 0.04 |

**Height**

| Parameter | Full | | | Free a,c=0 | | | a=1, free c | | | a=1, c=0 (best) | | |
|---|---|---|---|---|---|---|---|---|---|---|---|---|
| | est | cilo | cihi | est | cilo | cihi | est | cilo | cihi | est | cilo | cihi |
| s | 0.69 | 0.33 | 1.05 | 0.57 | 0.55 | 0.59 | 0.70 | 0.31 | 1.08 | 0.57 | 0.55 | 0.59 |
| h | 0.78 | 0.37 | 1.19 | 0.96 | 0.89 | 1.02 | 0.76 | 0.34 | 1.18 | 0.93 | 0.89 | 0.96 |
| m | 0.16 | 0.14 | 0.18 | 0.16 | 0.14 | 0.18 | 0.15 | 0.13 | 0.16 | 0.15 | 0.13 | 0.16 |
| r | 0.55 | 0.50 | 0.59 | 0.55 | 0.50 | 0.60 | 0.55 | 0.50 | 0.59 | 0.55 | 0.50 | 0.60 |
| a | 0.97 | 0.92 | 1.02 | 0.97 | 0.92 | 1.02 | | | | | | |
| c | 0.16 | | 0.51 | | | | 0.15 | -0.19 | 0.50 | | | |
| Rgpartner | 0.10 | 0.00 | 0.17 | 0.14 | 0.11 | 0.18 | 0.09 | 0.00 | 0.14 | 0.13 | 0.11 | 0.15 |
| Rgsibling | 0.55 | 0.50 | 0.59 | 0.55 | 0.50 | 0.59 | 0.55 | 0.50 | 0.59 | 0.55 | 0.50 | 0.59 |
| Rginlaw | 0.05 | 0.00 | 0.10 | 0.08 | 0.06 | 0.10 | 0.05 | 0.00 | 0.08 | 0.07 | 0.06 | 0.08 |
| Rgcoinlaw | 0.01 | 0.00 | 0.02 | 0.01 | 0.01 | 0.02 | 0.00 | 0.00 | 0.01 | 0.01 | 0.01 | 0.01 |

**Depression**

| Parameter | Full | | | Free a,c=0 (best) | | | a=1, free c | | | a=1, c=0 | | |
|---|---|---|---|---|---|---|---|---|---|---|---|---|
| | est | cilo | cihi | est | cilo | cihi | est | cilo | cihi | est | cilo | cihi |
| s | 0.41 | 0.04 | 0.77 | 0.26 | 0.21 | 0.32 | 0.40 | 0.06 | 0.74 | 0.27 | 0.21 | 0.33 |
| h | 0.34 | 0.03 | 0.65 | 0.53 | 0.39 | 0.67 | 0.28 | 0.04 | 0.52 | 0.41 | 0.32 | 0.51 |
| m | 0.28 | 0.20 | 0.35 | 0.27 | 0.20 | 0.35 | 0.17 | 0.16 | 0.19 | 0.17 | 0.16 | 0.19 |
| r | 0.65 | 0.42 | 0.87 | 0.70 | 0.50 | 0.90 | 0.66 | 0.43 | 0.88 | 0.71 | 0.51 | 0.91 |
| a | 0.79 | 0.68 | 0.91 | 0.80 | 0.69 | 0.91 | | | | | | |
| c | 0.13 | -0.05 | 0.30 | | | | 0.07 | -0.05 | 0.18 | | | |
| Rgpartner | 0.03 | 0.00 | 0.13 | 0.08 | 0.03 | 0.11 | 0.01 | 0.00 | NA | 0.03 | 0.02 | 0.04 |
| Rgsibling | 0.65 | 0.51 | 0.87 | 0.70 | 0.48 | 0.89 | 0.66 | 0.51 | 0.88 | 0.71 | 0.49 | 0.89 |
| Rginlaw | 0.02 | 0.00 | 0.09 | 0.05 | 0.03 | 0.10 | 0.01 | 0.00 | 0.03 | 0.02 | 0.01 | 0.03 |
| Rgcoinlaw | 0.00 | 0.00 | 0.01 | 0.00 | 0.00 | 0.01 | 0.00 | 0.00 | 0.00 | 0.00 | 0.00 | 0.00 |

Notes: s = sqrt(genetic signal); h = sqrt(heritability); m = mating; r = correlation in genetic signal between siblings; a = alpha (correlation between mated and measured phenotype); f = shared family environment.

**Supplementary Note 2. Polygenic scoring method, adjustment for principal components, and sensitivity analyses using different thresholds**

Polygenic scores were calculated using the following parameters (set via the relevant flags in the PRSice software):

- Clumping parameters: window size = 500kb; p-value threshold = 1; r2 threshold= 0.25
- Minor allele frequency: 1%
- Exclusion of MHC region specified at chr6:25000000-34000000

Supplementary Table 7 describes the correlations between partners in polygenic scores by the threshold for including a SNP in the polygenic score. For educational attainment and height, using higher cut-offs implied higher correlations between partners' polygenic scores, but this levelled off around p=0.0005. For depression, the partners' polygenic scores had low correlations at all thresholds.

**Supplementary Table 7.** Partner correlation in polygenic scores by threshold for including SNPs.

| | p=5e-8 | p=5e-7 | p=5e-6 | p=5e-5 | p=0.0005 | p=0.005 | p=0.05 | p=0.50 | p=1 |
|---|---|---|---|---|---|---|---|---|---|
| Education | 0.039 | 0.052 | 0.065 | 0.080 | 0.093 | 0.109 | 0.118 | 0.117 | 0.116 |
| Height | 0.050 | 0.053 | 0.054 | 0.059 | 0.063 | 0.067 | 0.074 | 0.084 | 0.084 |
| Depression | 0.001 | 0.011 | 0.004 | 0.005 | 0.009 | 0.006 | 0.006 | 0.003 | 0.003 |

Notes: p-values on the x-axis are not results of this study, but refer to the probability of observing an association between a SNP and the phenotype of equal or greater magnitude in the discovery GWAS under the hypothesis of no association. The p-values are not adjusted for multiple testing; $p < 5*10^{-8}$ is considered genomewide significant.

To evaluate the influence of different methods for adjusting for principal components, we calculated associations between relatives' polygenic scores and relatives' phenotypes using three methods. These principal components are described in more detail in Supplementary Methods, page 1. The three sets of correlations between relatives are shown in Supplementary Table 8 and 9. In the first set of results (a), we estimated crude associations between polygenic scores without taking the principal components into account. In the second set of results (b), we first residualized polygenic scores and phenotypes on the principal components and then estimated the associations between these residuals. In the third set of results (c), we included the principal components directly in the model used to estimate the correlations. In Supplementary Table 8, we present results adjusted for 20 principal components, which is the number of principal components used in our analyses. Supplementary Table 9 we present results adjusted for 50 principal components to check if this changes the results. The correlations were estimated in OpenMx, and the principal components were set to influence the means in the model. The results indicate that the three methods for estimating the correlations provide similar results, with differences in the second decimal place. Most differences were 0.00 or 0.01, with the largest being 0.03 (correlations between siblings' polygenic scores for height). We consider these differences to be trivial and of no practical importance. The small consequences of adjusting for the principal components may be due to the homogeneity among participants in the Norwegian Mother, Father and Child Cohort Study (MoBa), from which we draw our sample.

We identified 1,492 pairs of second, third, or fourth-degree relatives in the sample by using the KING (Kinship-based INference for Gwas) software (https://www.kingrelatedness.com/) with the -ibs command (identity by state). Siblings (first-degree relatives) were already a part of the design. As a sensitivity analysis, we randomly excluded one individual from each pair and recalculated the correlations between relatives' polygenic scores and relatives' phenotypes. This led to changes in correlations of at most 0.01 compared to Supplementary Table 8.

**Supplementary Table 8.** Correlations between relatives' polygenic scores and relatives' phenotypes estimated in three ways: a) crude; b) residualized on 20 principal components; and c) by including 20 principal components (PC) for each relative in the model.

| **Educational attainment** | | | | | | | | | |
|---|---|---|---|---|---|---|---|---|---|
| Correlation | Unadjusted | | | Residualised, 20 PCs | | | Adjusted for 20 PCs | | |
| | est | cilo | cihi | est | cilo | cihi | est | cilo | cihi |
| Polygenic score, partners | 0.12 | 0.11 | 0.13 | 0.11 | 0.10 | 0.12 | 0.11 | 0.11 | 0.11 |
| Polygenic score, siblings | 0.55 | 0.52 | 0.57 | 0.55 | 0.52 | 0.57 | 0.56 | 0.53 | 0.58 |
| Polygenic score, inlaws | 0.06 | 0.03 | 0.09 | 0.06 | 0.03 | 0.09 | 0.06 | 0.05 | 0.06 |
| Polygenic score, coinlaws | 0.07 | 0.02 | 0.11 | 0.06 | 0.01 | 0.10 | 0.06 | 0.01 | 0.11 |
| Phenotype, partners | 0.42 | 0.42 | 0.43 | 0.40 | 0.39 | 0.41 | 0.40 | 0.40 | 0.41 |
| Phenotype, siblings | 0.40 | 0.39 | 0.42 | 0.37 | 0.33 | 0.40 | 0.37 | 0.36 | 0.40 |
| Phenotype, inlaws | 0.29 | 0.28 | 0.31 | 0.29 | 0.26 | 0.32 | 0.27 | 0.24 | 0.30 |
| Phenotype, coinlaws | 0.19 | 0.17 | 0.21 | 0.20 | 0.16 | 0.25 | 0.21 | 0.16 | 0.25 |
| **Height** | | | | | | | | | |
| Correlation | Unadjusted | | | Residualised, 20 PCs | | | Adjusted for 20 PCs | | |
| | est | cilo | cihi | est | cilo | cihi | est | cilo | cihi |
| Polygenic score, partners | 0.07 | 0.06 | 0.09 | 0.05 | 0.03 | 0.06 | 0.05 | 0.05 | 0.07 |
| Polygenic score, siblings | 0.56 | 0.53 | 0.58 | 0.52 | 0.49 | 0.55 | 0.55 | 0.52 | 0.57 |
| Polygenic score, inlaws | 0.05 | 0.01 | 0.08 | 0.03 | 0.00 | 0.06 | 0.04 | 0.03 | 0.04 |
| Polygenic score, coinlaws | 0.05 | 0.00 | 0.10 | 0.03 | -0.02 | 0.07 | 0.02 | -0.03 | 0.08 |
| Phenotype, partners | 0.16 | 0.15 | 0.16 | 0.15 | 0.13 | 0.17 | 0.16 | 0.16 | 0.17 |
| Phenotype, siblings | 0.49 | 0.48 | 0.51 | 0.46 | 0.41 | 0.49 | 0.48 | 0.44 | 0.52 |
| Phenotype, inlaws | 0.08 | 0.06 | 0.10 | 0.06 | 0.01 | 0.10 | 0.06 | 0.05 | 0.07 |
| Phenotype, coinlaws | 0.03 | 0.00 | 0.06 | 0.04 | -0.02 | 0.11 | 0.05 | -0.02 | 0.11 |
| **Depression** | | | | | | | | | |
| Correlation | Unadjusted | | | Residualised, 20 PCs | | | Adjusted for 20 PCs | | |
| | est | cilo | cihi | est | cilo | cihi | est | cilo | cihi |
| Polygenic score, partners | 0.01 | -0.01 | 0.02 | 0.00 | -0.01 | 0.01 | 0.00 | 0.00 | 0.01 |
| Polygenic score, siblings | 0.53 | 0.50 | 0.55 | 0.52 | 0.50 | 0.55 | 0.54 | 0.51 | 0.56 |
| Polygenic score, inlaws | 0.01 | -0.03 | 0.04 | 0.00 | -0.03 | 0.03 | 0.00 | -0.03 | 0.01 |
| Polygenic score, coinlaws | 0.02 | -0.03 | 0.07 | 0.01 | -0.04 | 0.06 | 0.02 | -0.03 | 0.07 |
| Phenotype, partners | 0.18 | 0.17 | 0.18 | 0.17 | 0.16 | 0.19 | 0.17 | 0.17 | 0.18 |
| Phenotype, siblings | 0.14 | 0.12 | 0.16 | 0.12 | 0.08 | 0.17 | 0.12 | 0.07 | 0.16 |
| Phenotype, inlaws | 0.05 | 0.04 | 0.07 | 0.05 | 0.01 | 0.08 | 0.04 | 0.03 | 0.04 |
| Phenotype, coinlaws | 0.04 | 0.02 | 0.06 | 0.05 | 0.00 | 0.10 | 0.04 | -0.01 | 0.09 |

**Supplementary Table 9.** Correlations between relatives' polygenic scores and relatives' phenotypes estimated in three ways: a) crude; b) residualized on 50 principal components; and c) by including 50 principal components (PC) for each relative in the model

| **Educational attainment** | | | | | | | | | |
|---|---|---|---|---|---|---|---|---|---|
| Correlation | Unadjusted | | | Residualised 50 PCs | | | Adjusted for 50 PCs | | |
| | est | cilo | cihi | est | cilo | cihi | est | cilo | cihi |
| Polygenic score, partners | 0.12 | 0.11 | 0.13 | 0.11 | 0.10 | 0.12 | 0.11 | NA | 0.12 |
| Polygenic score, siblings | 0.55 | 0.52 | 0.57 | 0.54 | 0.52 | 0.57 | 0.56 | 0.53 | 0.58 |
| Polygenic score, inlaws | 0.06 | 0.03 | 0.09 | 0.06 | 0.03 | 0.09 | 0.06 | 0.05 | 0.06 |
| Polygenic score, coinlaws | 0.07 | 0.02 | 0.11 | 0.06 | 0.01 | 0.10 | 0.06 | 0.01 | 0.11 |
| Phenotype, partners | 0.42 | 0.42 | 0.43 | 0.40 | 0.39 | 0.41 | 0.40 | 0.40 | 0.40 |
| Phenotype, siblings | 0.40 | 0.39 | 0.42 | 0.37 | 0.33 | 0.40 | 0.37 | 0.33 | 0.40 |
| Phenotype, inlaws | 0.29 | 0.28 | 0.31 | 0.29 | 0.26 | 0.32 | 0.27 | NA | 0.27 |
| Phenotype, coinlaws | 0.19 | 0.17 | 0.21 | 0.20 | 0.16 | 0.25 | 0.20 | 0.16 | 0.25 |
| **Height** | | | | | | | | | |
| Correlation | Unadjusted | | | Residualised 50 PCs | | | Adjusted for 50 PCs | | |
| | est | cilo | cihi | est | cilo | cihi | Est | cilo | Cihi |
| Polygenic score, partners | 0.07 | 0.06 | 0.09 | 0.05 | 0.04 | 0.06 | 0.05 | 0.05 | 0.05 |
| Polygenic score, siblings | 0.56 | 0.53 | 0.58 | 0.52 | 0.49 | 0.55 | 0.55 | 0.52 | 0.57 |
| Polygenic score, inlaws | 0.05 | 0.01 | 0.08 | 0.03 | 0.00 | 0.06 | 0.04 | 0.03 | 0.04 |
| Polygenic score, coinlaws | 0.05 | 0.00 | 0.10 | 0.02 | -0.03 | 0.06 | 0.02 | -0.03 | 0.07 |
| Phenotype, partners | 0.16 | 0.15 | 0.16 | 0.15 | 0.14 | 0.17 | 0.16 | NA | NA |
| Phenotype, siblings | 0.49 | 0.48 | 0.51 | 0.46 | 0.41 | 0.49 | 0.49 | 0.45 | 0.53 |
| Phenotype, inlaws | 0.08 | 0.06 | 0.10 | 0.06 | 0.02 | 0.11 | 0.07 | 0.06 | 0.08 |
| Phenotype, coinlaws | 0.03 | 0.00 | 0.06 | 0.04 | -0.02 | 0.10 | 0.04 | -0.03 | 0.11 |
| **Depression** | | | | | | | | | |
| Correlation | Unadjusted | | | Residualised 50 PCs | | | Adjusted for 50 PCs | | |
| | est | cilo | cihi | est | cilo | cihi | est | cilo | cihi |
| Polygenic score, partners | 0.01 | -0.01 | 0.02 | 0.00 | -0.01 | 0.01 | 0.00 | 0.00 | 0.00 |
| Polygenic score, siblings | 0.53 | 0.50 | 0.55 | 0.52 | 0.50 | 0.55 | 0.54 | 0.51 | 0.56 |
| Polygenic score, inlaws | 0.01 | -0.03 | 0.04 | 0.00 | -0.03 | 0.03 | 0.00 | -0.03 | 0.00 |
| Polygenic score, coinlaws | 0.02 | -0.03 | 0.07 | 0.01 | -0.03 | 0.06 | 0.01 | -0.04 | 0.06 |
| Phenotype, partners | 0.18 | 0.17 | 0.18 | 0.17 | 0.16 | 0.19 | 0.17 | 0.17 | 0.18 |
| Phenotype, siblings | 0.14 | 0.12 | 0.16 | 0.12 | 0.08 | 0.17 | 0.11 | 0.07 | 0.15 |
| Phenotype, inlaws | 0.05 | 0.04 | 0.07 | 0.05 | 0.01 | 0.08 | 0.04 | 0.04 | 0.05 |
| Phenotype, coinlaws | 0.04 | 0.02 | 0.06 | 0.05 | 0.00 | 0.10 | 0.04 | -0.01 | 0.09 |

Supplementary Fig. 7 presents model parameters from rGenSi models using polygenic scores with different thresholds, whereas Supplementary Fig. 8 similarly presents genetic correlations between relatives derived from these models. Using higher cut-offs was associated with increases in the genetic signal for all phenotypes and smaller confidence intervals for the rGenSi estimated genetic correlations. Otherwise, the results remained similar across thresholds.

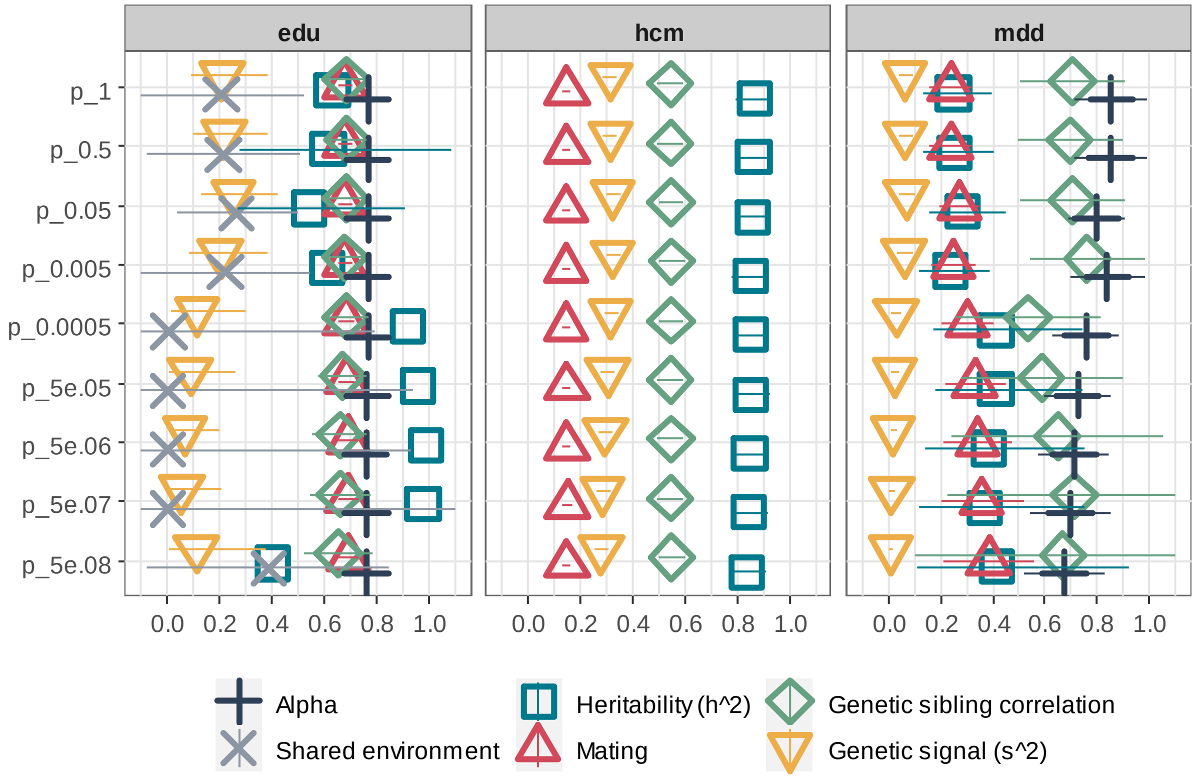


**Supplementary Fig. 7. Parameter estimates by thereshold for including SNPs in polygenic scores.** Estimated parameters from rGenSi models using polygenic scores with different cut-offs for inclusion of SNPs, including 95% likelihood-based confidence intervals. p-values on y-axis refer to the probability of observing an association between a SNP and the phenotype of equal or greater magnitude in the discovery GWAS under the hypothesis of no association. The p-values are not adjusted for multiple testing; $p < 5*10^{-8}$ is considered genomewide significant. edu=educational attainment at age 30; hcm = height in centimeters; mdd = symptoms of major depressive disorder. Based on genotype data from *n*=26,681 complete pairs of partners, *n*=2,170 complete sibling pairs, *n*=3,905 complete in-law pairs, and *n*=1,763 complete co-in-law pairs, and phenotype data from *n*=63,781 complete pairs of partners, *n*=13,455 complete sibling pairs, *n*=21,496 complete in-law pairs, and *n*=8,699 complete co-in-laws pairs.

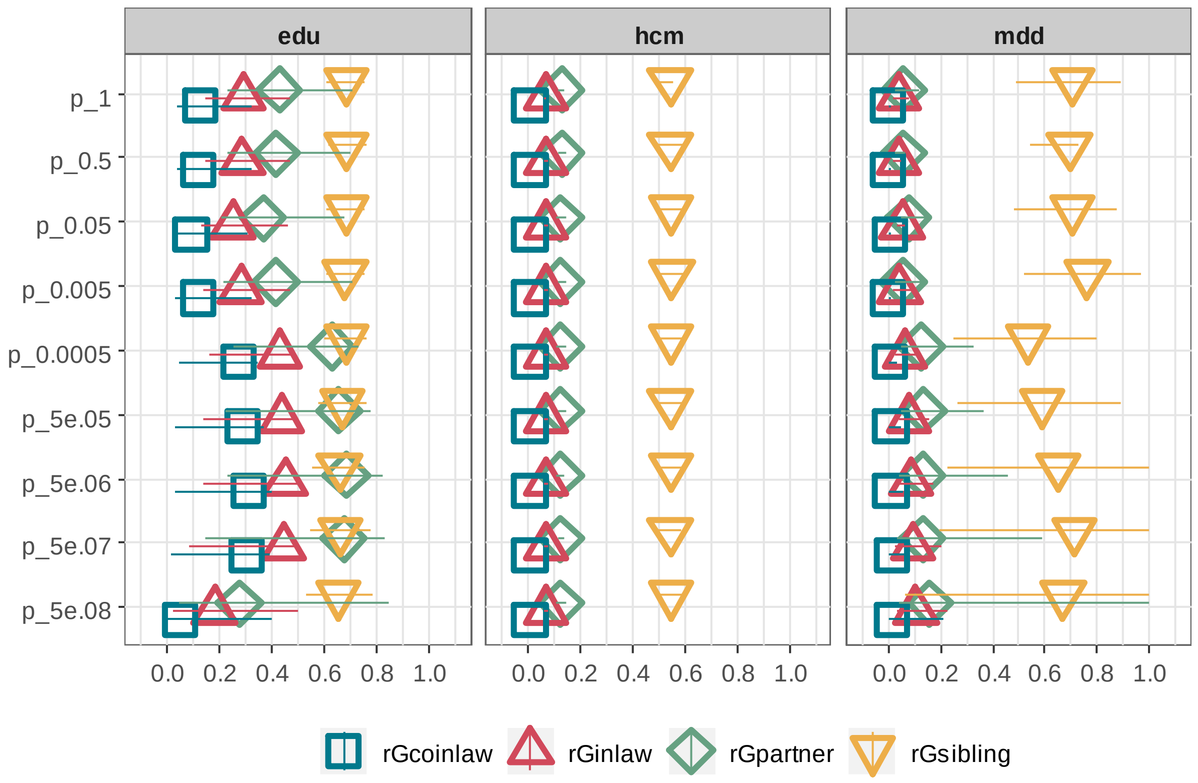


**Supplementary Fig. 8. Genetic correlations by thereshold for including SNPs in polygenic scores.** Estimated genetic correlations from rGenSi models using polygenic scores with different cut-offs for inclusion of SNPs, including 95% likelihood-based confidence intervals. p-values on y-axis refer to the probability of observing an association between a SNP and the phenotype of equal or greater magnitude in the discovery GWAS under the hypothesis of no association. The p-values are not adjusted for multiple testing; $p < 5*10^{-8}$ is considered genomewide significant. edu=educational attainment at age 30; hcm = height in centimeters; mdd = symptoms of major depressive disorder. Based on genotype data from *n*=26,681 complete pairs of partners, *n*=2,170 complete sibling pairs, *n*=3,905 complete in-law pairs, and *n*=1,763 complete co-in-law pairs, and phenotype data from *n*=63,781 complete pairs of partners, *n*=13,455 complete sibling pairs, *n*=21,496 complete in-law pairs, and *n*=8,699 complete co-in-laws pairs.

**Supplementary References**


1. Cloninger C. R. Interpretation of intrinsic and extrinsic structural relations by path analysis: theory and applications to assortative mating. *Genetical Research* **36**, 133-145, 10.1017/s0016672300019765 (1980).

2. Balbona J. V., Kim Y. & Keller M. C. Estimation of Parental Effects Using Polygenic Scores. *Behav Genet* **51**, 264-278, 10.1007/s10519-020-10032-w (2021).

3. Robinson M. R.*, et al.* Genetic evidence of assortative mating in humans. *Nature Human Behaviour* **1**, 0016, 10.1038/s41562-016-0016 (2017).

4. Keller M. C.*, et al.* Modeling extended twin family data I: description of the Cascade model. *Twin Res Hum Genet* **12**, 8-18, 10.1375/twin.12.1.8 (2009).
