# Supplementary material for: Modeling assortative mating and genetic similarities between partners, siblings, and in-laws
Source: Nat Commun. 2022 Mar 1;13:1108. doi: 10.1038/s41467-022-28774-y (PMC8888605; doi:10.1038/s41467-022-28774-y)
Supplement: Supplementary file 3 — Description of Additional Supplementary Files [file 41467_2022_28774_MOESM3_ESM.pdf]

Description of Additional Supplementary Files

File Name: Supplementary Software 1
Description: R language script to simulate assortative mating across multiple generations and fit the Correlations in Genetic Signal (rGenSi) model.
