# Supplementary material for: Modeling assortative mating and genetic similarities between partners, siblings, and in-laws
Source: Nat Commun. 2022 Mar 1;13:1108. doi: 10.1038/s41467-022-28774-y (PMC8888605; doi:10.1038/s41467-022-28774-y)
Supplement: Supplementary file 5 — Reporting Summary [file 41467_2022_28774_MOESM5_ESM.pdf]

nature portfolio

Corresponding author(s): Torvik, Fartein Ask

Last updated by author(s): Jan 18, 2022

Reporting Summary

Nature Portfolio wishes to improve the reproducibility of the work that we publish. This form provides structure for consistency and transparency in reporting. For further information on Nature Portfolio policies, see our Editorial Policies and the Editorial Policy Checklist.

Statistics

For all statistical analyses, confirm that the following items are present in the figure legend, table legend, main text, or Methods section.

n/a	Confirmed	
☐	☒	The exact sample size (n) for each experimental group/condition, given as a discrete number and unit of measurement
☐	☒	A statement on whether measurements were taken from distinct samples or whether the same sample was measured repeatedly
☐	☒	The statistical test(s) used AND whether they are one- or two-sided<br>*Only common tests should be described solely by name; describe more complex techniques in the Methods section.*
☐	☒	A description of all covariates tested
☐	☒	A description of any assumptions or corrections, such as tests of normality and adjustment for multiple comparisons
☐	☒	A full description of the statistical parameters including central tendency (e.g. means) or other basic estimates (e.g. regression coefficient) AND variation (e.g. standard deviation) or associated estimates of uncertainty (e.g. confidence intervals)
☐	☒	For null hypothesis testing, the test statistic (e.g. F, t, r) with confidence intervals, effect sizes, degrees of freedom and P value noted<br>*Give P values as exact values whenever suitable.*
☒	☐	For Bayesian analysis, information on the choice of priors and Markov chain Monte Carlo settings
☒	☐	For hierarchical and complex designs, identification of the appropriate level for tests and full reporting of outcomes
☐	☒	Estimates of effect sizes (e.g. Cohen's d, Pearson's r), indicating how they were calculated

Our web collection on statistics for biologists contains articles on many of the points above.

Software and code

Policy information about availability of computer code

Data collection	No software was used for data collection.
Data analysis	R 4.0.2; OpenMx 2.19.1; EIGENSTRAT version 6.1.4; Shapeit2 release 837; PRSice; KING (Kinship-based INference for Gwas).<br><br>Script developed for this paper can be found here: https://osf.io/v9ybu/

For manuscripts utilizing custom algorithms or software that are central to the research but not yet described in published literature, software must be made available to editors and reviewers. We strongly encourage code deposition in a community repository (e.g. GitHub). See the Nature Portfolio guidelines for submitting code & software for further information.

Data

Policy information about availability of data

All manuscripts must include a data availability statement. This statement should provide the following information, where applicable:

- Accession codes, unique identifiers, or web links for publicly available datasets
- A description of any restrictions on data availability
- For clinical datasets or third party data, please ensure that the statement adheres to our policy

MoBa data can be accessed by application to the Regional Committee for Medical and Health Research Ethics in Norway and MoBa (https://www.fhi.no/en/studies/moba/for-forskere-artikler/research-and-data-access/). The consent given by the participants does not open for storage of data on an individual level in repositories or journals.

Field-specific reporting

Please select the one below that is the best fit for your research. If you are not sure, read the appropriate sections before making your selection.

☐ Life sciences ☒ Behavioural & social sciences ☐ Ecological, evolutionary & environmental sciences

For a reference copy of the document with all sections, see nature.com/documents/nr-reporting-summary-flat.pdf

Behavioural & social sciences study design

All studies must disclose on these points even when the disclosure is negative.

Study description	Population based pregnancy cohort. Observational quantitative data.
Research sample	The study is based on the Norwegian Mother, Father and Child Cohort study. The target population of the study is all women who give birth in Norway and their partners. There are no exclusion criteria. Studies of assortative mating need large samples of the general population, which this cohort study provided. The sample is largely representative for the Norwegian population, however, parents with high educational attainment were more likely to participate, which may reduce generalizability. The sample is homogenous and mostly of European ancestry, potentially constraining generalizability to similar populations.
Sampling strategy	All hospitals and maternity units with more than 100 births annually, altogether 52 units, are to be included, and by January 2006, 50 units participate in the study. For practical reasons, the sampling frame comprises pregnant women who attend routine ultrasound examination. Together with appointments for ultrasound scanning in week 17–18 of pregnancy, the pregnant women receive a postal invitation that includes an informed consent form, the first questionnaire, an information brochure as well as consent form and questionnaire for the father.
Data collection	When she attends the ultrasound examination, the woman is asked whether she has consented to participate. If yes, the woman is referred to the laboratory for blood and urine samples and, if he consents, also a blood sample from the father. If a woman or a couple decides to participate in the study while attending the ultrasound examination, a consent form can be filled out there and then. All filled-in questionnaires are sent by mail to a central facility where they are registered, scanned, and verified.<br><br>The questionnaires were filled in at home - we can therefore not confirm if the participant was alone or if anyone else was present when they were filled in.<br><br>The hypotheses were not conceived at the time of data collection - the researchers at that time were therefore blinded to the hypotheses. The authors of this paper were not blinded to the hypotheses.<br><br>Educational attainment at age 30 were gathered from governmental registers administered by Statistics Norway.<br><br>Height in centimeters was self reported by mothers and fathers.<br><br>Symptoms of major depressive disorders were self-reported by mothers and fathers using the Lift-Time hHistory of Major Depression questionnaire, which is based on DSM-III.
Timing	Cohort recruited from July 1999 until December 2008. Data collection is still going on in the cohort. Genotyped 2012-2021.
Data exclusions	We used data for all individuals with valid and with currently available genomic data. Genotyping is underway for all participants.
Non-participation	The total participation rate for all invited pregnancies is 41% (112908/277702). Individuals who declined to participate were not asked to state a reason for this. Later, 146 families (0.1% of the recruited) withdrew their consent and had their data deleted (they did not have to state a reason for this). As we use data from the initial questionnaire, non-participation in later follow-up questionnaires is not relevant.
Randomization	No randomization. Polygenic scores and phenotypes were adjusted for 20 principal components to account for population stratification, and up to 50 principal components in sensitivity analyses.

Reporting for specific materials, systems and methods

We require information from authors about some types of materials, experimental systems and methods used in many studies. Here, indicate whether each material, system or method listed is relevant to your study. If you are not sure if a list item applies to your research, read the appropriate section before selecting a response.

Materials & experimental systems

n/a	Involved in the study
☒	☐ Antibodies
☒	☐ Eukaryotic cell lines
☒	☐ Palaeontology and archaeology
☒	☐ Animals and other organisms
☐	☒ Human research participants
☒	☐ Clinical data
☒	☐ Dual use research of concern

Methods

n/a	Involved in the study
☒	☐ ChIP-seq
☒	☐ Flow cytometry
☒	☐ MRI-based neuroimaging

Human research participants

Policy information about studies involving human research participants

Population characteristics	See above
Recruitment	The study is based on voluntary participation. Informed consent was obtained from all study participants. It is likely that there is a socioeconomic gradient that influences prevalence estimates. However, the aim of MoBa is to provide valid estimates of associations between putative causal factors and disease, and both the prevalence of exposures and diseases may be different from what is found in the total population, but the estimate of association can still be valid.
Ethics oversight	The Regional Committees for Medical and Health Research Ethics, Southern and Eastern Norway (project# 2017/2205)

Note that full information on the approval of the study protocol must also be provided in the manuscript.
